# Lag‐sensitive cross‐correlation analysis of mother–infant HF‐HRV synchrony during free interaction: An exploratory feasibility study

**DOI:** 10.14814/phy2.71116

**Published:** 2026-09-22

**Authors:** Aoi Sakurai, Kunihiro Koide, Yoshiyuki Kasahara, Yasuka Nakamura, Toyoko Yoshizawa

**Affiliations:** ^1^ Department of Child Health Nursing Tohoku University Graduate School of Medicine Sendai Japan; ^2^ Department of Maternal and Fetal Therapeutics Tohoku University Graduate School of Medicine Sendai Japan; ^3^ Department of Nursing Yamagata Prefectural University of Health Sciences Yamagata Japan; ^4^ Mie Prefectural College of Nursing Tsu Mie Japan

**Keywords:** cross‐correlation, heart rate variability, mother–infant interaction, synchrony, time lag

## Abstract

Caregiver–infant interaction involves coordinated behavioral and physiological processes. Heart rate variability (HRV) may provide a physiological index of these interactions. This study preliminarily evaluated an analytical approach for characterizing mother–infant HRV synchrony with temporal lags. Electrocardiograms were simultaneously recorded during free interaction. Thirty sessions were obtained from 15 mother–infant dyads, and 16 analyzable sessions from 10 unique dyads were included in the final analysis. Normalized cross‐correlation analysis of high‐frequency HRV was performed using MATLAB R2022b. Highly correlated intervals accounted for a mean of 75% of the analyzed interaction time, and positive and negative lags occurred in nearly equal proportions. Observed synchrony exceeded the session‐specific surrogate distribution in 13 of 16 sessions based on the empirical p‐value criterion. These findings support the feasibility of a lag‐sensitive, surrogate‐informed approach for preliminarily describing putative mother–infant HRV coordination during free interaction.

## 1 INTRODUCTION

### 1.1 Parent–child interaction as the foundation of attachment

The parent–child relationship in mammals is fundamentally organized through attachment (Bowlby, 1969). In humans, attachment provides a theoretical framework for understanding the reciprocal behavioral and emotional regulation between caregivers and infants. This framework was originally formalized by John Bowlby, who proposed that early disruptions in caregiver–infant proximity may lead to separation anxiety and long‐term alterations in socioemotional development. Bowlby further described attachment as a developmental process that progresses through multiple stages, culminating in the formation of an internal working model that guides subsequent interpersonal interaction.

Building on this theoretical foundation, Mary Ainsworth introduced the Strange Situation Procedure, a standardized observational paradigm that enables systematic investigation of how caregivers function as a secure base (Ainsworth & Bell, 1970). This approach provided one of the earliest empirical frameworks for quantifying individual differences in attachment‐related behaviors (Klaus & Kennell, 1977). Subsequent studies in developmental psychology have expanded these methodologies by incorporating diverse behavioral and environmental measures, thereby reinforcing and refining the theoretical constructs proposed by Bowlby (Jethava et al., 2022).

Despite these advances, existing attachment assessments rely predominantly on behavioral observations that may be influenced by observational context. Although attachment disorders have been extensively discussed in psychiatry, diagnostic, and intervention frameworks applicable across developmental stages remain insufficiently established (Zimmermann & Soares, 2019). Furthermore, reliable epidemiological data are limited, highlighting the value of complementary approaches for characterizing processes occurring during parent–child interactions.

### 1.2 Heart rate variability as a physiological index of interaction

Recent research has increasingly examined physiological coordination between parents and children as an additional dimension of dyadic interaction (Brazelton et al., 1975; Feldman, 2017; Matsumoto et al., 2010; Papousek, 1995). Traditional approaches primarily examine observable interactions such as physical contact and exchanges of vocal and emotional signals. However, these measures may not fully capture underlying regulatory dynamics.

In this context, heart rate variability (HRV), derived from electrocardiogram (ECG) signals, has emerged as a promising physiological index for quantifying autonomic regulation in parent–child dyads. HRV provides information on autonomic modulation and offers a means to characterize physiological dynamics beyond observable behaviors. By integrating physiological signals with interaction analysis, HRV‐based approaches may provide complementary information about physiological coordination during parent–child interactions.

HRV refers to beat‐to‐beat variation in the R‐R interval (RRI), defined as the interval between successive R waves on the ECG and measured in milliseconds. Previous studies have examined physiological synchrony between parents and children using HRV‐based measures, with particular attention to respiratory sinus arrhythmia (RSA) (Abney et al., 2021; Porges, 1991). In addition, previous studies have examined the concordance of cardiac vagal tone between mothers and infants, highlighting the developmental aspects of physiological coupling (Bornstein & Suess, 2000). The theoretical grounding for this study is also informed by Polyvagal Theory (Porges, 1995, 2007), which posits that the vagal system plays a critical role in the dynamic regulation of physiological states and in social engagement. This framework has been instrumental in understanding autonomic co‐regulation in close interpersonal relationships, particularly between caregivers and infants. RSA is associated with parasympathetic modulation and reflects heart‐rate fluctuations related to the respiratory cycle. As RSA is associated with physiological states related to social interaction and emotional regulation, it serves as a valuable indicator for assessing physical and mental health and stress response. In measurement analyses, RSA is commonly quantified using high‐frequency heart rate variability (HF‐HRV), corresponding to power within the HF component of HRV and widely used in studies of parent–child physiological synchrony. Recent meta‐analytic work has further shown that mother–child RSA synchrony is a meaningful but context‐dependent phenomenon (Miller et al., 2023).

In the present study, we focused on HF‐HRV because it is associated with RSA and has been widely used in studies of parent–infant physiological coordination. However, HF‐HRV is also influenced by respiratory rate and depth. Because respiration was not measured or controlled in the present study, HF‐HRV was used to characterize temporal physiological coordination between mothers and infants rather than as a respiration‐controlled measure of RSA or vagal activity.

### 1.3 Previous analytical approaches to HRV synchrony

HRV synchrony refers to temporal similarity between HRV patterns of two individuals. Previous studies have reported physiological synchrony between closely related individuals, including parents and children. A previous study reported similar HRV patterns between pregnant women and their fetuses (Widatalla et al., 2022). Additionally, HRV synchronization has been observed in other intimate relationships, such as between partners in a marital relationship and between dogs and their owners (Gates et al., 2015) (Katayama et al., 2019). Physiological synchrony has been examined in relation to interactive behaviors such as contact, eye contact, and verbal exchanges.

Despite the accumulation of reports on HRV synchronization, technical challenges persist in this area of research (Capraz et al., 2023). Previous studies suggest that physiological synchrony may involve temporal offsets that vary according to individual physiological and interactional characteristics, making these temporal patterns challenging to characterize. Previous approaches have often estimated RSA or HRV using values averaged over fixed time windows (Abney et al., 2021). Such approaches can characterize physiological activity over the measurement interval but may not capture temporal offsets between two physiological signals. Cross‐correlation analysis provides an approach for examining temporal correspondence between two time series while allowing potential time lags to be considered (Dean & Dunsmuir, 2016). Therefore, an analytical approach that accounts for temporal shifts may be useful for characterizing temporal physiological coordination (Dean & Dunsmuir, 2016; Van Egeren et al., 2001).

However, averaging HF components fails to capture aspects of the RSA itself and may result in the loss of important information regarding the dynamic regulation of individual reactivity and emotion. This consideration is particularly relevant given that behaviors such as eye contact between parents and children can change within seconds.

To date, only a few studies have examined methods for analyzing temporal shifts in synchronization. For example, (Abney et al., 2021) used cross‐correlation analysis to examine RSA coupling of infants and their mothers in a continuous time series. This study demonstrated that cross‐correlation analysis can effectively reveal HRV synchronization.

(Capraz et al., 2023) hypothesized that HRV synchronization occurs during parent–child interactions, suggesting scenarios in which the mother precedes the infant and vice versa. They conducted an analysis that considered temporal lags within ±3 s. However, a detailed investigation of the lag‐window settings has not yet been conducted, and the analysis method has not been sufficiently verified to accurately capture actual temporal lags.

Therefore, this study aimed to develop and preliminarily evaluate a cross‐correlation‐based method for characterizing mother–infant HRV synchrony with temporal lag during free interactions. We further explored whether synchrony patterns exceeded those expected from chance‐level temporal alignment using a surrogate‐based comparison.

## 2 METHODS

### 2.1 Ethical considerations

This study was conducted in accordance with the Declaration of Helsinki and approved by the Ethics Committee of the Tohoku University Graduate School of Medicine (2022‐1‐910). Written informed consent was obtained from the mothers for their own participation and for their infants' participation in the study. All technical images were anonymized prior to submission.

### 2.2 Participants

The participants were 15 mother–infant dyads, including healthy infants aged 5–7 months and their mothers, who were recruited between December 2022 and March 2023. Participants were recruited through advertisements in local childcare newspapers and social media, and potential participants completed an online recruitment form. A full description of the study was provided at the time of application. Eligible participants included infants born at 36–41 weeks of gestation and mothers who were at least 20 years old and able to read and write in Japanese. The exclusion criteria included serious psychiatric symptoms, such as severe cognitive dysfunction or depression; significant skin diseases in the child; and cases in which a physician, midwife, or nurse deemed participation to be inappropriate.

The same experimental procedure was conducted twice for each of the 15 mother–infant dyads, yielding 30 sessions. For the final analysis, we used only continuous ECG segments with uninterrupted maternal and infant data, in which R peaks could be reliably detected after artifact removal and paired HRV time series could be synchronized. In four dyads later labeled as D1–D4 in Table S1, transient device errors occurred immediately after the start of recording; therefore, the subsequent continuous analyzable segments obtained after the recording stabilized were used for analysis. Of the 30 recorded sessions, 16 met the data quality criteria and were included in the final analysis, whereas 14 were excluded. The reasons for exclusion were device recording failure (*n* = 5), session interruption (*n* = 2), and electrode detachment or excessive artifacts due to infant movement (*n* = 7). The final analytic dataset comprised 16 sessions from 10 unique dyads; six dyads contributed two sessions, and four dyads contributed one session. Details of data quality screening and session inclusion status are provided in Table S1.

### 2.3 Experimental procedure

Data were collected between December 2022 and March 2023 in a rented studio in Sendai City, Miyagi Prefecture, Japan. Prior to data collection, the participants were informed via email that mothers and infants with fever or cold symptoms should refrain from participation. Their health conditions were also checked on site. On arrival, the mothers were encouraged to feed their infants and change their diapers to ensure that the infants were calm during the recording sessions. Both the mother and child were fitted with ECG monitors and allowed a few minutes of free interaction to acclimatize to the electrodes before the ECG monitoring began. Before the study commenced, the mothers and children spent time on a yoga mat, and measurements were taken for 5 min. Each dyad was scheduled for two recording sessions approximately 2 weeks apart under the same free‐interaction protocol. Free play was defined as activities in which mothers freely interacted with their children by talking, touching, and playing with them.

### 2.4 Measures

The collected data included participant demographics and electrocardiographic findings. The following attributes were recorded: maternal age, gestational age at delivery, family type, employment status, highest level of education, and infant sex. ECG signals were recorded using a BioLog DL‐5500 system with wireless DL‐500 sensors at a sampling frequency of 1 kHz. Sensors were positioned at the upper sternum of the mothers and near the clavicular region of the infants using medical‐grade adhesive. This system was selected because its small wireless sensors allowed relatively unrestricted recording in infants.

### 2.5 HRV signal processing

ECG signals were processed using a custom MATLAB‐based ECG cleaning program that allows automated QRS detection and manual correction. The raw files contained maternal ECG, infant ECG, and synchronization‐marker channels. The program generated cleaned RRI time‐series files and corresponding QRS‐detection and cleaning records for each mother and infant.

Because the recording system had already filtered the ECG signals, no additional hum or band‐pass filter was applied before QRS detection. The ECG waveform was enhanced using a second‐order difference filter. The MATLAB findpeaks function was then applied to the absolute value of the second‐order difference waveform, and candidate QRS complexes were automatically detected using a level‐trigger algorithm with amplitude‐ and time‐domain thresholds adjusted appropriately for the maternal and infant ECG recordings. Because ECG amplitude varied across recordings, amplitude‐related thresholds were adjusted on a recording‐by‐recording basis when necessary. R‐peak positions were identified on the raw ECG waveform, and peak polarity was aligned according to the predominant polarity identified during automated detection. Candidate artifacts were automatically flagged during MATLAB‐based ECG processing, and final artifact classification was confirmed by visual inspection of the ECG waveform and RRI series rather than by a single fixed numerical threshold.

After automated detection and artifact flagging, all retained ECG recordings were visually inspected and edited by the first author. When necessary, R peaks were manually added or deleted, and artifact classifications were revised based on visual inspection of the ECG waveform and RRI series. Ambiguous cases were additionally reviewed together with another member of the research team. Artifact‐flagged RRIs, including suspected ectopic beats and nonphysiological RRI values, were stored as missing values (NaN). No interpolation was performed for these missing RRIs, and the corresponding observations were not used in subsequent spectral estimation.

R‐peak detection was considered reliable when detected peaks consistently corresponded to visually identifiable QRS complexes and the resulting RRI time series showed physiologically plausible beat‐to‐beat intervals without abrupt nonphysiological changes. Segments with ambiguous R peaks, unstable electrode contact, excessive movement artifacts, or insufficient continuous analyzable data were excluded.

To quantify signal quality in the retained data, the proportion of artifact‐flagged or removed RRIs was calculated within the actual analysis intervals used for cross‐correlation analysis and summarized in Table S2. R peaks that were manually corrected and retained in the cleaned RRI series were not counted as artifact‐flagged or removed RRIs. Across the 16 retained sessions, the session‐level proportion of artifact‐flagged or removed RRIs was 0.29% ± 0.60% for mothers and 0.02% ± 0.08% for infants.

**FIGURE 1:**
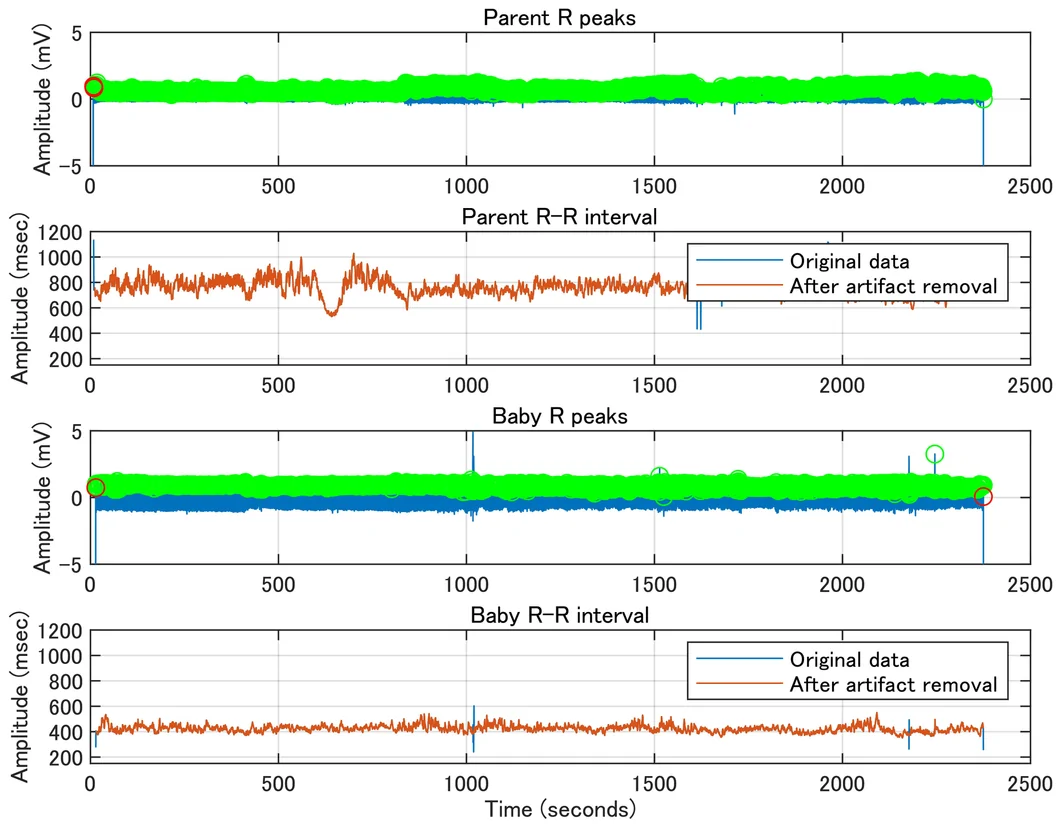
Representative RRI time‐series signals derived from ECG data for a mother–infant pair after QRS detection, artifact screening, and manual review. The upper two panels show maternal ECG‐derived R peaks and the cleaned maternal RRI time series; the lower two panels show the corresponding infant signals. Five minutes (300 s) of data were used when a complete session was retained; for sessions with shorter analyzable intervals, the corresponding continuous analysis window was used. These RRI time series were used for the frequency‐domain analysis shown in Figure 2.

The RRI time series was subjected to spectral analysis using the plomb function in MATLAB, and HF‐HRV power was calculated by integrating spectral power within the predefined high‐frequency band. The Lomb–Scargle approach was used because it accommodates unequally spaced RRI observations, including missing values introduced by artifact removal. Analysis was conducted in 30‐s windows advanced in 0.5‐s increments. The predefined HF band was 0.15–0.40 Hz for mothers and 0.24–1.04 Hz for infants (Bar‐Haim et al., 2000). HF‐HRV power was expressed in msec^2^ and used in the subsequent cross‐correlation analyses (Figure 1).

**FIGURE 2:**
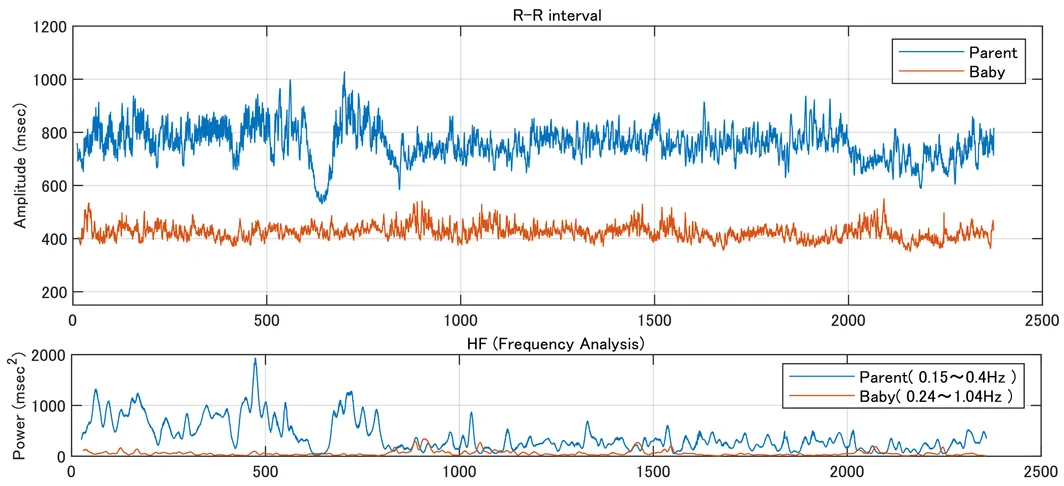
Representative RRI and HF‐HRV time‐series data. The upper panel shows the RRI time series from a mother–infant pair. The mother's RRI time series is shown in blue, and the infant's RRI time series is shown in orange. The lower panel shows HF‐HRV power derived from spectral analysis of the RRI time series using the Lomb–Scargle periodogram implemented with the plomb function in MATLAB. HF‐HRV was calculated as integrated power within the predefined high‐frequency band and is expressed in msec^2^. The mother's HF‐HRV power is shown in blue, and the infant's HF‐HRV power is shown in orange. These HF‐HRV time‐series values were used for the subsequent cross‐correlation analyses.

### 2.6 Statistical analysis

Normalized cross‐correlation analysis of the maternal and infant HF‐HRV time series was performed using MATLAB R2022b (CC analysis). The calculated coefficients ranged from −1 to 1, with positive coefficients indicating similar temporal fluctuations and negative coefficients indicating inverse temporal fluctuations (Dean & Dunsmuir, 2016). In this study, only positive cross‐correlation coefficients of 0.8 or higher were used as an operational definition of high cross‐correlation, based on the conventional interpretation that correlation coefficients of 0.8–1.0 indicate a strong positive association (Swinscow & Campbell, 1997). Negative cross‐correlation coefficients, including values of −0.8 or lower, were not included in the synchrony index because this study focused on positive HRV synchrony during mother–infant interaction. However, this cutoff should be regarded as a practical and exploratory threshold rather than an absolute physiological criterion.

**FIGURE 3:**
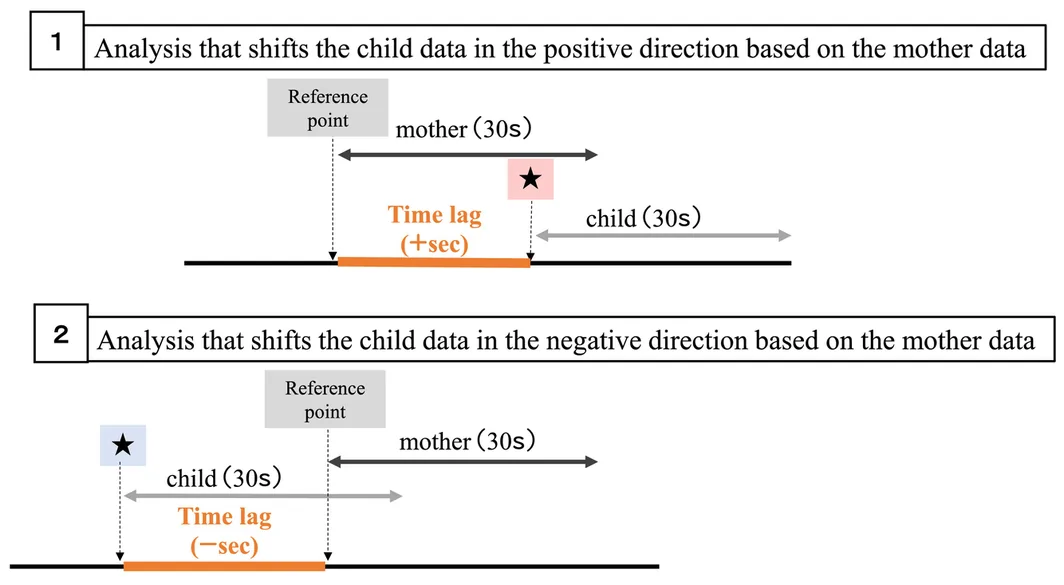
Schematic illustration of the cross‐correlation procedure used to identify synchronization points. For each 30‐s maternal reference window, the infant HF‐HRV window was shifted within the predefined lag range of −30 to +30 s. Positive peaks were detected using the predefined criteria, including *r* ≥ 0.8, minimum prominence 0.01, and minimum separation 10 s. If multiple eligible peaks were detected, the peak with the highest cross‐correlation coefficient was selected as the synchronization point.

The analytical procedure is outlined below and illustrated schematically in Figure 3. A stepwise pseudocode of the complete signal‐processing and synchrony‐analysis workflow is provided in Methods S1. Statistical summaries were generated at the session level. Because repeated sessions from the same dyads were not statistically independent, session‐level results were treated as descriptive and exploratory. A dyad‐level sensitivity analysis was also conducted by averaging repeated session values within each dyad.

#### 2.6.1 Analysis 1: Analysis with the infant data shifted positively relative to the mother data

1. Using the mother data as a reference, cross‐correlation was obtained by shifting the infant data positively from the starting point of the analysis.
2. Positive peaks in the cross‐correlation sequence were identified using the MATLAB islocalmax function with MinProminence set to 0.01 and MinSeparation set to 10 s. Peaks with cross‐correlation coefficients ≥0.8 were considered eligible.
3. When multiple eligible peaks were identified, the peak with the highest cross‐correlation coefficient was selected.
4. The selected peak was adopted as the synchronization point, and the corresponding time deviation from the starting analysis point was recorded as the lag (time lag).
5. This procedure was repeated at 0.5‐s intervals.

Specifically, a 30‐s window was defined for the mother data, while the infant windows were shifted within a + 30 s interval from the analysis start point. This window size was deemed sufficient for frequency analysis because, at 0.15 Hz (the lower limit of the maternal HF component range), it included at least 4 cycles within one window. The lag search range was selected as an exploratory setting to capture potential temporal offsets in mother–infant HF‐HRV synchrony. However, this range should not be interpreted as a definitive physiological window.

#### 2.6.2 Analysis 2: Analysis of the infant data shifted negatively relative to the mother data

1. Cross‐correlation was obtained by shifting the child's data negatively from the starting point of the analysis, with the mother's data serving as the reference. In Analysis 1, the examination was performed by shifting the data in the +30 s interval; this study performed the same shifting analysis in the −30 s interval.
2. Steps 2–5 were conducted in the same manner as in Analysis 1.

**FIGURE 4:**
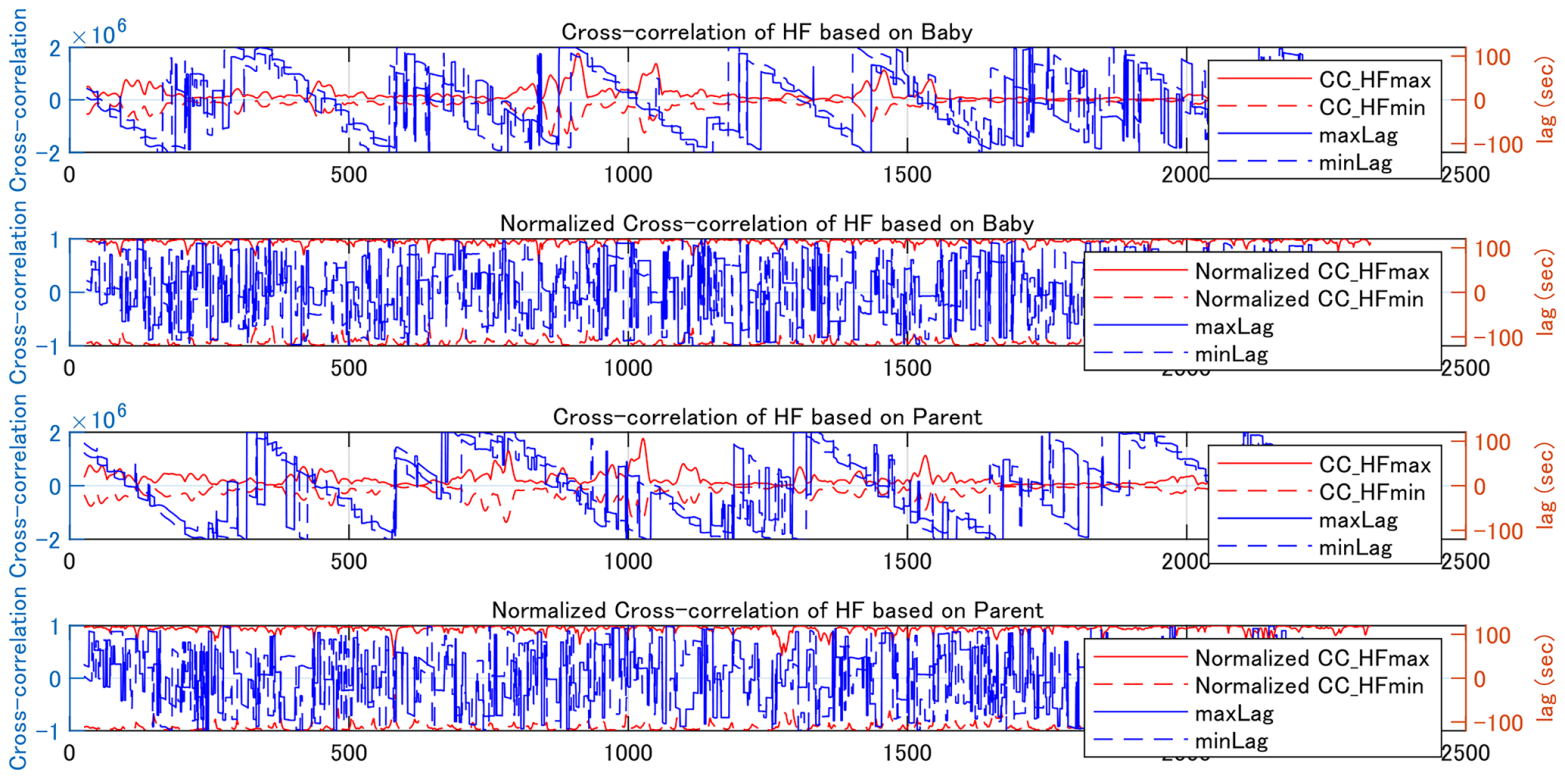
Example graph of cross‐correlation analysis results. Graphical overview of the normalized cross‐correlation analysis using maternal and infant HF‐HRV power. The horizontal axis represents time (s). The cross‐correlation and lag traces illustrate the output from which eligible positive peaks were identified using the predefined criteria. The selected synchronization points were used to calculate the highly correlated time and lag‐category results presented in Table 2.

Figure 4 illustrates a graph of the results of Analyses 1 and 2 for a mother–infant pair in the study. When both analyses were performed, eligible peaks on both sides of zero were compared, and the peak with the larger cross‐correlation coefficient was selected. No exact ties for the highest coefficient occurred in the analyzed data; under the software implementation, an exact tie would retain the first peak returned by the peak‐selection routine. The selected synchronization points were designated as Lag+, Lag−, and Lag0. In the Lag+ case, the infant's HF‐HRV pattern aligned with the mother's after a positive temporal offset, whereas in the Lag− case, the mother's HF‐HRV pattern aligned with that of the infant after a negative temporal offset. Each reference window was assigned to no more than one lag category, and windows without an eligible peak were not classified as highly correlated. This study examined both positive and negative time shifts to characterize the temporal dynamics of HRV synchronization in mother–infant interactions.

For the dyad‐level sensitivity analysis, session‐level values were averaged within each dyad, yielding one value for each of the 10 unique dyads. This analysis included the proportion of highly correlated time, the Lag+, Lag−, and Lag0 proportions, and the observed–surrogate synchrony difference.

The selected lag was classified as Lag+, Lag−, or Lag0 according to its sign, so each reference window was assigned to no more than one lag category.

### 2.7 Surrogate‐based evaluation of HRV synchrony

#### 2.7.1 Theoretical background and analytical rationale

Physiological synchrony indices derived from time‐series data may be inflated by autocorrelation within individual signals and shared measurement intervals, leading to spurious synchrony, even in the absence of genuine dyadic interaction. This issue is particularly relevant for HRV signals that exhibit strong temporal dependencies (Task Force of the European Society of Cardiology and the North American Society of Pacing and Electrophysiology, 1996). Consequently, cross‐correlation‐based measures alone are insufficient to distinguish meaningful interpersonal coupling from coincidental temporal alignment.

To address this limitation, surrogate‐based testing has been used in synchrony research to evaluate whether observed temporal alignment exceeds that expected under a specified null model. Previous studies have reported physiological synchrony in parent–child interactions (Davis et al., 2018), while methodological studies have highlighted the limitations of time‐series correlation analyses and the value of surrogate‐based evaluation (Ramseyer & Tschacher, 2011; Theiler et al., 1992).

#### 2.7.2 Surrogate data generation

For sessions with analyzable durations shorter than 300 s, the surrogate comparison was restricted to complete 30‐s blocks, and any remaining partial block was excluded. Surrogate datasets were generated by independently permuting the temporal order of 30‐s segments within the maternal and infant HF‐HRV time series, thereby disrupting their original temporal correspondence. This block‐permutation procedure retained local temporal structure within each 30‐s segment but did not fully preserve long‐range autocorrelation or temporal dependencies across segment boundaries.

For each session, 100 independently block‐permuted surrogate pairs were generated and analyzed using the same cross‐correlation procedure as the observed data. Observed and surrogate synchrony indices were calculated using the corresponding analysis‐window denominator for each session. Specifically, the observed synchrony index was defined as highly correlated time divided by the corresponding analysis‐window duration, whereas the surrogate synchrony index was calculated as the number of highly correlated 0.5‐s points divided by the number of 0.5‐s points in the corresponding surrogate analysis window. For every session, the surrogate mean, surrogate SD, surrogate 95th percentile, observed–surrogate mean difference, and empirical p‐value were calculated. The empirical *p*‐value was defined as *p* = (1 + the number of surrogate values greater than or equal to the observed value)/101.

#### 2.7.3 Decision criteria relative to the block‐permuted null model

Evidence that observed synchrony exceeded that expected under the block‐permuted null model was assessed using the empirical surrogate distribution. A session was considered to exceed the block‐permuted surrogate distribution when the empirical *p*‐value was <0.05, corresponding to an observed value in the upper tail of its session‐specific surrogate distribution. The session‐level results are reported in Table S4.

As a dyad‐level sensitivity analysis, session‐level observed synchrony, surrogate means, and observed–surrogate differences were averaged within dyad; single‐session dyads retained their session‐level values. These results are reported in Table S5.

The use of 100 surrogate iterations limited the resolution of the empirical distribution but was retained because each iteration required a complete session‐level cross‐correlation analysis and substantially greater iteration counts were not computationally feasible. Accordingly, the surrogate analysis was treated as an exploratory comparison with a single block‐permuted null model rather than as definitive evidence of dyad‐specific physiological coupling. Because the present procedure evaluated a single null model based on 30‐s block permutations, additional null models, including circular shifts, phase‐randomized surrogates, non‐partner dyad pairing, session‐level permutation, and alternative block lengths, should be examined in future studies.

## 3 RESULTS

### 3.1 Overview of HRV synchrony during free interaction

The basic attributes of the analytic sample are presented in Table 1. Of the 15 mother–infant dyads initially enrolled, 10 unique dyads were included in the final analysis after excluding recordings with device recording failure, session interruption, electrode detachment, or excessive movement artifacts. The parents' mean age was 32.8 ± 3.8 years, with a range of 25–39 years. Among the three infants born at 36–38 weeks of gestation, one was born at 36 weeks and two were born at 38 weeks; the remaining seven infants were born at 39–41 weeks. The analytic sample included two boys and eight girls. Educational attainment included two parents with high school diplomas, one with junior college or vocational school education, and seven with bachelor's or higher degrees. Employment status included maternity leave for seven participants, return to work for one, and unemployment for two.

**TABLE 1:** Participant characteristics of the analytic sample (*N* = 10).

Variable	Category	*N*
Parents' age	25–29 years	2
	30–34 years	4
	35–39 years	4
Gestational age at delivery	36–38 weeks	3
	39–41 weeks	7
Family type	Nuclear family	9
	Extended family	1
Infant sex	Male	2
	Female	8
Current employment status	On maternity leave	7
	Returned to work	1
	Unemployed	2
Educational background	High school graduate	2
	Junior college/vocational school	1
	Bachelor's degree or higher	7

*Note*: The 36–38‐week category included one infant born at 36 weeks and two infants born at 38 weeks.

### 3.2 Cross‐correlation analysis of mother–infant HRV

Table 2 shows the intervals during which high positive cross‐correlations, defined as coefficients ≥0.8, were observed during the analysis period. For each analyzed session, the proportion of highly correlated time was calculated by dividing the duration of highly correlated intervals by the total analyzed time. Across the 16 analyzed sessions, the session‐level proportion of highly correlated intervals ranged from 60.7% to 91.7%, with a mean of 74.5% ± 9.0%, rounded to 75%.

**TABLE 2:** Distribution of highly correlated time across lag categories.

ID	Dyad	Session	Analyzed time (s)	Highly correlated time (s)	Lag − time (s)	Lag − proportion	Lag + time (s)	Lag + proportion	Lag0 time (s)	Lag0 proportion
1	D1	1	225.45	180.0	141.0	0.78	37.5	0.21	1.5	0.01
2	D2	1	211.31	169.5	112.0	0.66	57.5	0.34	0.0	0.00
3	D3	1	247.07	165.0	54.5	0.33	103.0	0.62	7.5	0.05
4	D4	1	196.16	145.5	41.0	0.28	102.5	0.70	2.0	0.01
5	D1	2	300.00	239.5	71.0	0.30	168.5	0.70	0.0	0.00
6	D2	2	300.00	208.5	122.5	0.59	84.0	0.40	2.0	0.01
7	D3	2	300.00	272.5	149.5	0.55	123.0	0.45	0.0	0.00
8	D4	2	300.00	275.0	140.0	0.51	133.5	0.49	1.5	0.01
9	D5	2	300.00	205.0	151.5	0.74	52.5	0.26	1.0	0.00
10	D6	1	300.00	197.0	122.5	0.62	74.5	0.38	0.0	0.00
11	D7	1	300.00	240.0	89.5	0.37	148.0	0.62	2.5	0.01
12	D8	1	300.00	224.5	145.0	0.65	78.5	0.35	1.0	0.00
13	D6	2	300.00	202.0	126.0	0.62	65.0	0.32	11.0	0.05
14	D8	2	300.00	182.0	110.5	0.61	71.0	0.39	0.5	0.00
15	D9	2	300.00	232.0	118.5	0.51	111.0	0.48	2.5	0.01
16	D10	1	300.00	196.5	113.5	0.58	82.5	0.42	0.5	0.00
**Total**	**—**	**—**	**4479.99**	**3334.5**	**1808.5**	**0.54**	**1492.5**	**0.45**	**33.5**	**0.01**

*Note*: Lag−, Lag+, and Lag0 proportions represent the proportion of each lag category within the highly correlated time period for each session. Therefore, the sum of the Lag−, Lag+, and Lag0 times equals the highly correlated time. The mean session‐level proportion of highly correlated times was 74.5%, rounded to 75%. The pooled time‐weighted proportion calculated from the total row was 74.4%. Bold values indicate the total or pooled values across all sessions.

### 3.3 Time‐lag characteristics of HRV synchronization

The proportions of the highly correlated times assigned to each lag classification are shown in Table 2. Lag−, Lag+, and Lag0 represent the subcategories of the total highly correlated time. Therefore, the sum of the Lag−, Lag+, and Lag0 times corresponded to the total highly correlated time for each analyzed session. The highly correlated intervals were categorized as Lag+, Lag−, or Lag0, and the proportion of each lag category was calculated within each highly correlated period.

Among the intervals classified as highly correlated, the mean session‐level proportions assigned to Lag+ and Lag− were 44.6% ± 15.0% and 54.4% ± 15.2%, respectively. The corresponding ranges were 20.8%–70.5% for Lag+ and 28.2%–78.3% for Lag−, respectively. Lag0 accounted for only a small proportion of highly correlated intervals, with a mean of 1.1% ± 1.6% and a range of 0.0%–5.5%.

### 3.4 Dyad‐level sensitivity analysis

As a sensitivity analysis addressing the non‐independence of repeated sessions, session‐level values were averaged within each dyad. The mean dyad‐level proportion of highly correlated time was 0.742 ± 0.065. Within highly correlated intervals, the mean proportions were 0.545 ± 0.116 for Lag−, 0.445 ± 0.115 for Lag+, and 0.010 ± 0.008 for Lag0. These estimates were similar to the session‐level estimates, indicating that the distribution of positive and negative lag patterns was not driven solely by dyads with two retained sessions. The dyad‐level observed–surrogate synchrony difference was 0.200 ± 0.061. Detailed results are provided in Tables S3 and S5.

### 3.5 Comparison of observed HRV synchrony with block‐permuted surrogates

**FIGURE 5:**
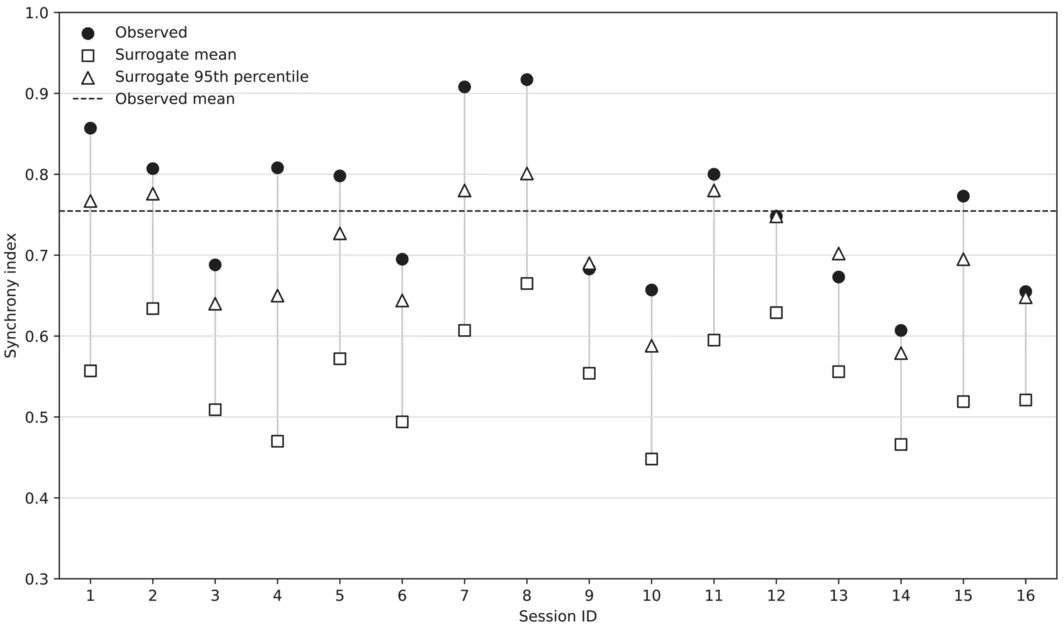
Session‐level observed synchrony relative to surrogate distributions. Filled circles indicate observed synchrony indices, open squares indicate surrogate means, and open triangles indicate session‐specific surrogate 95th percentiles. The dashed horizontal line indicates the mean observed synchrony index across the 16 sessions. Values are shown for the 16 analyzed sessions using the corresponding analysis‐window denominator for each session. Observed synchrony was considered to exceed the corresponding block‐permuted surrogate distribution when the empirical *p*‐value was <0.05. Under this criterion, observed synchrony exceeded the surrogate distribution in 13 of 16 sessions.

Using the corresponding analysis‐window denominator for each session, the mean observed synchrony index across the 16 sessions was 0.755 ± 0.094, compared with a mean surrogate value of 0.550 ± 0.065. The mean observed–surrogate difference was 0.205 ± 0.070. Based on the empirical *p*‐value criterion, observed synchrony exceeded the session‐specific surrogate distribution in 13 of 16 sessions, with empirical *p*‐values ranging from 0.010 to 0.109 across all sessions. Thus, observed synchrony exceeded the corresponding block‐permuted surrogate distribution in most, but not all, of the analyzed sessions (Figure 5; Table S4).

At the dyad level, the observed synchrony mean was 0.749 ± 0.075 and the surrogate mean was 0.549 ± 0.028, yielding a mean observed–surrogate difference of 0.200 ± 0.061. The positive dyad‐level difference was consistent across the 10 dyads, although the small sample and exploratory surrogate procedure warrant cautious interpretation.

## 4 DISCUSSION

### 4.1 Interpretation of temporal HRV coordination

The present findings suggest that high HRV synchrony was frequently observed across the analyzed sessions, with highly correlated intervals accounting for approximately 75% of the total interaction times. In our analysis of mother–infant HRV, we applied a lag‐sensitive sliding window method in which windows of the same width were shifted to both preceding and succeeding positions from the analysis start point. This approach allowed us to characterize temporal shifts in HRV synchrony. We found that these temporal shifts occurred at similar levels in both positive and negative lag directions, indicating that temporal HF‐HRV alignment was observed in both lag directions within the predefined search range. Physiological co‐regulation has been conceptualized as a core mechanism of adaptive caregiver–child interactions. In this context, Polyvagal Theory highlights the role of vagal pathways in supporting physiological synchrony and biobehavioral co‐regulation between caregivers and children (Porges, 1995, 2007). Consistent with this perspective, previous studies have reported associations between caregiver–child HRV coupling and emotional attunement or regulatory processes. These theoretical perspectives provide a context for examining physiological coordination during caregiver–infant interaction. However, the present findings should be interpreted as temporal HF‐HRV coordination rather than as direct evidence of vagal co‐regulation, behavioral regulation, or attachment‐related processes. Given that HRV is a minimally invasive and objective indicator, it may provide a basis for future studies examining the physiological aspects of parent–child interactions, although its clinical applicability remains to be established.

Lag+ represented intervals in which the infant's HF‐HRV pattern aligned with the mother's after a positive temporal offset (Capraz et al., 2023). Previous studies have suggested that changes in maternal HRV during parent–infant interactions may be associated with corresponding changes in infant HRV, even in early infancy. Lag+ therefore represented temporal alignment at a positive offset relative to the maternal signal. The present study also identified Lag− intervals in which the mother's HRV rhythm was aligned with that of the infant after a temporal offset. This result suggests that synchrony patterns were not limited to intervals in which maternal signals preceded infant signals. However, HF‐HRV alignment was observed with both positive and negative temporal offsets. These findings indicate that temporal HF‐HRV alignment was not restricted to one lag direction within the predefined search range. The experimental context should also be considered when interpreting the distribution of lag types. Because the sessions were conducted in a free‐play environment, there were no constraints on the occurrence of Lag+ or Lag− during the sessions. The varying proportions of Lag+ and Lag− across sessions suggest that each mother–infant pair may show a relative bias toward one lag direction over a limited period. At the same time, the aggregated results showed similar overall proportions of Lag+ and Lag− within the predefined lag‐search range. Future research incorporating behavioral observation will be important for clarifying which interactional events are associated with the occurrence of the Lag+ and Lag− groups.

However, because synchronized behavioral coding was not conducted in the present study, the observed HRV synchrony cannot yet be linked to specific interactive behaviors, such as eye contact, vocalization, touch, posture, movement, crying, or infant affect. Therefore, the present findings should be interpreted as temporal patterns of physiological coordination rather than direct evidence of specific behavioral or attachment‐related processes.

### 4.2 Methodological contributions and analytical considerations

A key contribution of this study is that temporal lag was explicitly incorporated into the evaluation of mother–infant HF‐HRV synchrony and examined against an empirical block‐permuted null distribution. Observed synchrony exceeded the session‐specific surrogate distribution in 13 of 16 sessions based on the empirical p‐value criterion, whereas the remaining sessions did not meet the empirical *p* < 0.05 criterion. At the dyad level, the observed–surrogate difference was positive across all 10 dyads and averaged 0.200 ± 0.061. This pattern indicates that observed HF‐HRV synchrony exceeded the present block‐permuted surrogate distribution in most, but not all, analyzed sessions. The findings therefore support the feasibility of the analytical framework under the present exploratory null model rather than establishing universal or dyad‐specific physiological coupling. The use of 30‐s windows also constrains the analysis of rapid adjustments; future studies should examine methods with finer temporal resolution.

### 4.3 Implications for parent–child interaction research

In the present study, highly correlated intervals accounted for a mean session‐level proportion of 74.5% (rounded to 75%) during free mother–infant interaction. Previous studies have generally reported lower or variable levels of synchrony in mother–infant interactions (Davis et al., 2018), reflecting the context‐dependent and dynamic nature of the physiological coupling.

One possible reason for this discrepancy may be that previous studies evaluated the average degree of synchronization and did not account for temporal offsets. In contrast, the present analysis used the maximum value within each interval as the cross‐correlation measure, which tends to produce higher values than the mean value. However, by incorporating temporal lag, the present method allowed HF‐HRV alignment to be characterized across positive and negative temporal offsets within the predefined search range (Abney et al., 2021). Additionally, developmental stage may influence patterns of physiological synchrony (Bar‐Haim et al., 2000). The early postnatal period may differ from later developmental stages in the temporal characteristics of mother–infant physiological coordination. Future studies focusing on different developmental stages may reveal variations in synchrony development as infants grow older.

A notable finding of this study was that Lag0 accounted for only 1% of the highly correlated intervals, whereas Lag+ and Lag− accounted for 99%. This result suggests that under the present analytical definition, mother–infant HRV synchrony during free interaction was rarely characterized by exact zero‐lag alignment and was more often observed with temporal offset. However, no directly comparable studies have examined this specific lag distribution, and the physiological significance of these temporal offsets is unclear. Therefore, lag information should be interpreted only as a temporal feature of HRV alignment and not as evidence of the underlying regulatory mechanisms.

Although Lag+ and Lag− represented positive and negative temporal offsets in HF‐HRV alignment, these lag categories should not be interpreted as evidence that one partner behaviorally led or regulated the other. Without synchronized behavioral coding, it remains unclear which interactive events, if any, are associated with these temporal‐lag patterns.

### 4.4 Characteristics of the subjects

The average age of the parents in the analytic sample was 32.8 years. National statistics in Japan indicate that the mean maternal age at childbirth in 2020 was 30.7, 32.7, and 33.8 years for the first, second, and third child, respectively. Thus, the parental age in the present analytic sample was broadly comparable to the national maternal age distribution (Ministry of Health, Labour and Welfare, Japan, 2022).

In terms of educational attainment, 7 of the 10 parents in the analytic sample had a bachelor's degree or higher. This was higher than the national proportion of women with college degrees (50.9%), suggesting that the study population may be slightly more educated than the general population (Gender Equality Bureau, Cabinet Office, Government of Japan, n.d.).

### 4.5 Limitations

This study was limited to infants aged 5–7 months and their mothers, which restricts the generalizability of our findings. Future research should examine physiological synchrony across a wider range of developmental stages and include father–infant dyads to validate the broader applicability of the proposed method.

Furthermore, although the sample size was modest, it aligns with prior studies examining physiological synchrony in mother–infant dyads, which typically include eight to 12 dyads (Davis et al., 2018). The dyad‐level sensitivity estimates were similar to the session‐level estimates, reducing concern that the descriptive pattern was driven only by dyads contributing two sessions. However, repeated sessions from the same dyads were not statistically independent; therefore, session‐level findings should be interpreted as descriptive and exploratory. In addition, overlapping 30‐s windows advanced in 0.5‐s steps produced substantial autocorrelation, and the effective sample size should be interpreted in relation to the 16 sessions and 10 unique dyads rather than the number of time‐series points.

The free‐play dyad‐only design also limits the causal and dyad‐specific interpretations of the observed synchrony. Because no non‐partner dyad pairing, resting baseline, no‐interaction condition, or still‐face condition was included, the present design cannot fully exclude synchrony attributable to shared environmental influences, common movement, shared posture, measurement artifacts, or nonspecific temporal structure.

Another limitation is the absence of synchronized behavioral coding in the study. Although the free‐play sessions included talking, touching, and toy play, specific behaviors such as eye contact, vocalization, touch, movement, crying, posture, and infant affect were not coded in synchrony with HRV signals. Therefore, physiological findings cannot yet be linked to specific mother–infant interactions. Future studies should combine lag‐sensitive HRV synchrony analysis with synchronized behavioral coding to clarify the behavioral events associated with HRV alignment and temporal lag patterns.

The surrogate analysis was based on 100 iterations of a single null model using random permutations of 30‐s segments. Because the surrogate block length was the same as the 30‐s analysis window used in the cross‐correlation procedure, local temporal structure within each block was retained. Therefore, this surrogate analysis should be interpreted as an exploratory comparison with a specific block‐permuted null model rather than as definitive evidence of dyad‐specific physiological coupling. The limited number of iterations also constrained the resolution of the empirical p‐values. Future studies should test robustness using more iterations where feasible and alternative null models, including circular shifts, phase‐randomized surrogates, non‐partner dyad pairing, session‐level permutation, and alternative block lengths.

The predefined ±30‐s lag range may also have influenced the probability of detecting high correlations and the resulting lag distribution. Because no additional lag‐window sensitivity analysis was performed, Lag+ and Lag− should be interpreted as descriptive temporal alignments within the predefined ±30‐s range rather than as established physiological lead–lag relationships. Sensitivity analyses using narrower and broader lag ranges are needed to evaluate the robustness of these patterns.

Respiration was not measured or controlled. Although HF‐HRV is associated with respiratory sinus arrhythmia and parasympathetic modulation, it is influenced by respiratory rate and depth (Task Force of the European Society of Cardiology and the North American Society of Pacing and Electrophysiology, 1996). Because respiratory characteristics differ between adults and infants (Bar‐Haim et al., 2000), and respiration may also vary during free interaction with vocalization, movement, and arousal, the present mother–infant HF‐HRV findings should not be interpreted as respiration‐controlled estimates of RSA or vagal activity.

Finally, as participation was limited to volunteers with internet access, the sample may have been biased toward caregivers with greater interest in parenting‐related research or greater access to the recruitment channels used in this study. This potential selection bias should be addressed in future studies that use diverse recruitment strategies to recruit participants. In addition, because 14 of the 30 recorded sessions were excluded primarily for technical or signal‐quality reasons, selection bias related to data retainability cannot be excluded.

### 4.6 Future directions

This study used normalized cross‐correlation analysis to characterize temporal HF‐HRV alignment between mothers and infants within a predefined lag range. Further research is needed to evaluate the robustness, physiological interpretation, and generalizability of these temporal patterns across different interaction contexts.

Future studies should include larger samples and examine whether comparable positive and negative lag patterns are observed in father–infant dyads. Future studies should also incorporate non‐partner pairing, shuffled mother–infant pairs, resting or neutral baseline conditions, and within‐versus between‐dyad comparisons to determine whether the observed HF‐HRV alignment is specific to interacting dyads or not. If these findings are replicated in larger samples, subsequent studies could examine whether temporal HF‐HRV patterns differ in populations with elevated psychosocial risk, such as mothers with postpartum depression.

## 5 CONCLUSION

This exploratory study demonstrated the feasibility of a lag‐sensitive HF‐HRV synchrony analysis for characterizing temporal coordination between mothers and infants during free interaction. Session‐ and dyad‐level estimates showed similar distributions of positive and negative lag patterns, and observed synchrony exceeded the corresponding block‐permuted surrogate distribution in 13 of 16 sessions. These findings remain preliminary because of the small analytic sample, the use of a single exploratory surrogate model with limited iterations, the absence of respiratory and synchronized behavioral measurements, and the lack of baseline or non‐partner comparison conditions.

## AUTHOR CONTRIBUTIONS

**Aoi Sakurai:** Conceptualization; data curation; formal analysis; investigation; methodology; visualization. **Kunihiro Koide:** Data curation; formal analysis; resources. **Yoshiyuki Kasahara:** Data curation; investigation; methodology. **Yasuka Nakamura:** Validation. **Toyoko Yoshizawa:** Conceptualization; funding acquisition; investigation; project administration; supervision.

## FUNDING INFORMATION

This work was supported by the Japan Society for the Promotion of Science (JSPS) KAKENHI [grant numbers 23K27893, 23H03203]; the Japan Science and Technology Agency (JST) SPRING [grant number JPMJSP2114]; and a collaborative research fund from Fam's Co., Ltd., Tokyo, Japan. Fam's Co., Ltd. provided financial support for the collaborative research but had no role in the study design, participant recruitment, data collection, data analysis, interpretation of the results, preparation of the manuscript, or the decision to submit the manuscript for publication.

## CONFLICT OF INTEREST STATEMENT

The authors declare no conflict of interest.

## ETHICS STATEMENT

This study was approved by the Ethics Committee of the Tohoku University Graduate School of Medicine (2022‐1‐910).

## CONSENT

Written informed consent was obtained from the mothers for their own participation and for their infants' participation.

## Supporting information


Data S1.
**Methods S1.** Stepwise pseudocode for ECG processing and HF‐HRV synchrony analysis.
**Table S1.** Data quality screening and session inclusion status.
**Table S2.** Proportion of artifact‐flagged or removed RRIs in retained sessions.
**Table S3.** Dyad‐level sensitivity analysis of highly correlated time and lag‐category proportions.
**Table S4.** Session‐level surrogate analysis using 100 block‐permuted surrogate time series.
**Table S5.** Dyad‐level observed–surrogate synchrony difference.

## Data Availability

The data that support the findings of this study are available from the corresponding author upon reasonable request. Due to ethical restrictions related to participant confidentiality, the datasets are not publicly available. *Code Availability Statement*: The MATLAB code used for signal processing and cross‐correlation analysis is not publicly deposited because it may be subject to institutional intellectual‐property and licensing considerations. However, the code is available from the corresponding author upon reasonable request.
