# Supplementary material for: Lag‐sensitive cross‐correlation analysis of mother–infant HF‐HRV synchrony during free interaction: An exploratory feasibility study
Source: Physiol Rep. 2026 Sep 22;14(18):e71116. doi: 10.14814/phy2.71116 (PMC13598298; doi:10.14814/phy2.71116)
Supplement: Supplementary file 1 [file PHY2-14-e71116-s001.docx]

**Supplementary Materials**

**Supplementary Methods S1. Stepwise pseudocode for ECG processing and HF-HRV synchrony analysis**

For each mother–infant session:

1. Import the simultaneously recorded maternal and infant ECG signals and synchronization markers.
2. Enhance the ECG waveform using a second-order difference filter. Apply the MATLAB *findpeaks* function to the absolute value of the second-order difference waveform and automatically detect QRS complexes using a level-trigger algorithm with amplitude- and time-domain thresholds adjusted separately for maternal and infant recordings as needed. As initial settings, set the QRS-enhancement filter coefficient to 33 for maternal ECG and 17 for infant ECG; the lower amplitude threshold to 10 μV; the amplitude-threshold coefficient to 30%; the upper heart-rate limit to 200 bpm for mothers and 300 bpm for infants; the time-threshold coefficient to 50%; and the peak-detection interval to 5 ms. Because ECG amplitude varies across recordings, adjust amplitude-related detection thresholds on a recording-by-recording basis when necessary. After automated detection, visually compare the detected QRS complexes with the raw ECG waveform. When visually identifiable QRS complexes are missed, adjust the detection threshold to increase sensitivity; when noise, movement-related deflections, or non-QRS waveform components are falsely detected, adjust the threshold to improve specificity. Repeat automated detection and threshold adjustment until visually identifiable QRS complexes are adequately detected without systematic false-positive detections. Identify the R-peak positions on the raw ECG waveform and align peak polarity according to the predominant polarity identified during automated detection. Apply the predominant polarity consistently within each recording.
3. Visually inspect all retained ECG recordings after automated QRS detection and artifact flagging. When a visually identifiable QRS complex is not detected automatically, manually add the corresponding R peak. When an automatically detected peak corresponds to noise, movement-related artifact, a non-QRS waveform component, or another spurious deflection rather than a true QRS complex, manually delete the detected peak. Review the resulting RRI time series together with the corresponding ECG waveform and revise artifact classifications when necessary. Additionally review ambiguous cases together with another member of the research team. Store artifact-flagged, suspected ectopic, and nonphysiological RRIs as NaN without interpolation. Exclude the corresponding observations from subsequent spectral estimation.
4. Calculate maternal and infant HF-HRV power from the RRI time series using Lomb–Scargle spectral analysis in 30-s windows advanced at 0.5-s intervals. Integrate spectral power over 0.15–0.40 Hz for mothers and 0.24–1.04 Hz for infants.
5. For each 30-s maternal reference window, calculate normalized cross-correlation while shifting the infant HF-HRV window within the predefined positive and negative lag ranges, corresponding to an overall lag-search range of −30 to +30 s.
6. Identify positive peaks in the cross-correlation sequence using the MATLAB islocalmax function with MinProminence set to 0.01 and MinSeparation set to 10 s. Retain peaks with cross-correlation coefficients ≥ 0.8.
7. If multiple eligible peaks are detected, select the peak with the highest cross-correlation coefficient. If eligible peaks occur in both positive- and negative-lag analyses, retain the peak with the higher coefficient. Classify the selected lag as Lag+, Lag−, or Lag0. Windows without an eligible peak are not classified as highly correlated.
8. Repeat the procedure at 0.5-s intervals and calculate the session-level synchrony index as the duration of highly correlated intervals divided by the corresponding analyzed duration. Calculate the proportions of Lag+, Lag−, and Lag0 within the highly correlated intervals.
9. Generate 100 surrogate mother–infant HF-HRV pairs per session by independently permuting the temporal order of 30-s segments within the maternal and infant time series. Apply the same cross-correlation and peak-selection procedure to each surrogate pair.
10. Calculate the surrogate mean, SD, 95th percentile, observed–surrogate difference, and empirical p-value for each session, where
    p = (1 + number of surrogate values ≥ observed value) / 101.
11. For the dyad-level sensitivity analysis, average session-level values within each dyad to obtain one value for each of the 10 unique dyads.

Note. Detailed signal-processing parameters and analytical procedures are described in Sections 2.5–2.7 of the main manuscript.

**Supplementary Table S1. Data quality screening and session inclusion status**

**A. Data quality screening**

| **Category** | **Number of sessions** |
| --- | --- |
| **Total recorded sessions** | 30 |
| Included sessions | 16 |
| Excluded sessions | 14 |
| Device recording failure | 5 |
| Session interruption | 2 |
| Electrode detachment or excessive artifacts due to infant movement | 7 |

**B. Session inclusion status among retained dyads**

| **Included sessions per dyad** | **Dyads** | **Number of dyads** | **Total included sessions** |
| --- | --- | --- | --- |
| 2 | D1, D2, D3, D4, D6, D8 | 6 | 12 |
| 1 | D5 (S2), D7 (S1), D9 (S2), D10 (S1) | 4 | 4 |
| Total | — | 10 | 16 |

Note. Exclusion categories were mutually exclusive. Device recording failure refers to failure to record, merge, or synchronize the paired maternal and infant ECG data required for HRV analysis.

**Supplementary Table S2. Proportion of artifact-flagged or removed RRIs in retained sessions**

**A. Session-level artifact/removed RRI proportions**

| **Dyad** | **Session** | **Mother (%)** | **Infant (%)** |
| --- | --- | --- | --- |
| D1 | 1 | 0.00% | 0.00% |
| D2 | 1 | 0.00% | 0.34% |
| D3 | 1 | 0.00% | 0.00% |
| D4 | 1 | 0.76% | 0.00% |
| D1 | 2 | 0.00% | 0.00% |
| D2 | 2 | 0.00% | 0.00% |
| D3 | 2 | 0.00% | 0.00% |
| D4 | 2 | 0.00% | 0.00% |
| D5 | 2 | 0.00% | 0.00% |
| D6 | 1 | 2.07% | 0.00% |
| D7 | 1 | 1.17% | 0.00% |
| D8 | 1 | 0.71% | 0.00% |
| D6 | 2 | 0.00% | 0.00% |
| D8 | 2 | 0.00% | 0.00% |
| D9 | 2 | 0.00% | 0.00% |
| D10 | 1 | 0.00% | 0.00% |

Note. Session-level percentages were calculated within the actual analysis interval used for cross-correlation analysis. Values are rounded to two decimal places.

**B. Summary across retained sessions**

| **Signal** | **Session-level mean ± SD** | **Median** | **Range** | **Pooled proportion across all RRIs** |
| --- | --- | --- | --- | --- |
| Mother | **0.29 ± 0.60%** | **0.00%** | **0.00–2.07%** | **0.33%** |
| Infant | **0.02 ± 0.08%** | **0.00%** | **0.00–0.34%** | **0.02%** |
| Mother and infant combined | **0.13 ± 0.23%** | **0.00%** | **0.00–0.75%** | **0.14%** |

Note. Calculations were restricted to the actual analysis intervals used for the cross-correlation analysis, each of which was 5 minutes or shorter. The pooled proportion was calculated by dividing the total number of artifact-flagged or removed RRIs by the total number of RRIs across all 16 sessions.

**Supplementary Table S3. Dyad-level sensitivity analysis of highly correlated time and lag-category proportions**

| **Dyad** | **Number of retained sessions** | **Highly correlated time / analyzed time** | **Lag− time / highly correlated time** | **Lag+ time / highly correlated time** | **Lag0 time / highly correlated time** |
| --- | --- | --- | --- | --- | --- |
| D1 | 2 | 0.798 | 0.540 | 0.456 | 0.004 |
| D2 | 2 | 0.749 | 0.624 | 0.371 | 0.005 |
| D3 | 2 | 0.788 | 0.439 | 0.538 | 0.023 |
| D4 | 2 | 0.829 | 0.395 | 0.595 | 0.010 |
| D5 | 1 | 0.683 | 0.739 | 0.256 | 0.005 |
| D6 | 2 | 0.665 | 0.623 | 0.350 | 0.027 |
| D7 | 1 | 0.800 | 0.373 | 0.617 | 0.010 |
| D8 | 2 | 0.678 | 0.627 | 0.370 | 0.004 |
| D9 | 1 | 0.773 | 0.511 | 0.478 | 0.011 |
| D10 | 1 | 0.655 | 0.578 | 0.420 | 0.003 |
| Mean ± SD |  | 0.742 ± 0.065 | 0.545 ± 0.116 | 0.445 ± 0.115 | 0.010 ± 0.008 |

Note. Repeated retained sessions were averaged within dyad; single-session dyads retained their session-level value. Lag-category proportions were calculated within highly correlated time.

**Supplementary Table S4. Session-level surrogate analysis using 100 block-permuted surrogate time series**

| **Dyad** | **Session** | **Observed synchrony** | **Surrogate mean** | **Surrogate SD** | **Surrogate 95th percentile** | **Empirical p-value** | **Observed − surrogate mean** |
| --- | --- | --- | --- | --- | --- | --- | --- |
| D1 | 1 | 0.857 | 0.557 | 0.118 | 0.767 | 0.010 | 0.300 |
| D2 | 1 | 0.807 | 0.634 | 0.114 | 0.776 | 0.030 | 0.173 |
| D3 | 1 | 0.688 | 0.509 | 0.085 | 0.640 | 0.010 | 0.179 |
| D4 | 1 | 0.808 | 0.470 | 0.106 | 0.650 | 0.010 | 0.338 |
| D1 | 2 | 0.798 | 0.572 | 0.098 | 0.727 | 0.030 | 0.226 |
| D2 | 2 | 0.695 | 0.494 | 0.094 | 0.644 | 0.040 | 0.201 |
| D3 | 2 | 0.908 | 0.607 | 0.100 | 0.780 | 0.010 | 0.301 |
| D4 | 2 | 0.917 | 0.665 | 0.081 | 0.801 | 0.010 | 0.252 |
| D5 | 2 | 0.683 | 0.554 | 0.085 | 0.690 | 0.069 | 0.129 |
| D6 | 1 | 0.657 | 0.448 | 0.085 | 0.588 | 0.010 | 0.209 |
| D7 | 1 | 0.800 | 0.595 | 0.092 | 0.780 | 0.020 | 0.205 |
| D8 | 1 | 0.748 | 0.629 | 0.080 | 0.748 | 0.069 | 0.119 |
| D6 | 2 | 0.673 | 0.556 | 0.085 | 0.702 | 0.109 | 0.117 |
| D8 | 2 | 0.607 | 0.466 | 0.080 | 0.579 | 0.030 | 0.141 |
| D9 | 2 | 0.773 | 0.519 | 0.092 | 0.695 | 0.010 | 0.254 |
| D10 | 1 | 0.655 | 0.521 | 0.084 | 0.648 | 0.040 | 0.134 |
| Mean ± SD | — | 0.755 ± 0.094 | 0.550 ± 0.065 | — | — | — | 0.205 ± 0.070 |

Note. For sessions with analyzable durations shorter than 300 s, the observed and surrogate synchrony indices used for the surrogate comparison were calculated using only complete 30-s blocks; any remaining partial block was excluded. The observed synchrony index was calculated as highly correlated time divided by the duration of the retained complete 30-s blocks. The surrogate synchrony index was calculated as the number of highly correlated 0.5-s points divided by the number of 0.5-s points in the corresponding surrogate analysis window. The empirical p-value was calculated as p = (1 + number of surrogate values greater than or equal to the observed value) / 101. Values were calculated using unrounded data and rounded to three decimal places.

**Supplementary Table S5. Dyad-level observed–surrogate synchrony difference**

| **Dyad** | **Number of retained sessions** | **Observed synchrony mean** | **Surrogate mean** | **Observed − surrogate mean** |
| --- | --- | --- | --- | --- |
| D1 | 2 | 0.828 | 0.565 | 0.263 |
| D2 | 2 | 0.751 | 0.564 | 0.187 |
| D3 | 2 | 0.798 | 0.558 | 0.240 |
| D4 | 2 | 0.863 | 0.567 | 0.295 |
| D5 | 1 | 0.683 | 0.554 | 0.129 |
| D6 | 2 | 0.665 | 0.502 | 0.163 |
| D7 | 1 | 0.800 | 0.595 | 0.205 |
| D8 | 2 | 0.678 | 0.547 | 0.130 |
| D9 | 1 | 0.773 | 0.519 | 0.254 |
| D10 | 1 | 0.655 | 0.521 | 0.134 |
| Mean ± SD |  | 0.749 ± 0.075 | 0.549 ± 0.028 | 0.200 ± 0.061 |

Note. Repeated retained sessions were averaged within dyad. Observed and surrogate synchrony indices were calculated using the corresponding analysis-window denominator for each session.
